# Personality traits of core self-evaluation as predictors on clinical decision-making in nursing profession

**DOI:** 10.1371/journal.pone.0233435

**Published:** 2020-05-18

**Authors:** Nikolina Farčić, Ivana Barać, Jadranka Plužarić, Vesna Ilakovac, Stana Pačarić, Zvjezdana Gvozdanović, Robert Lovrić

**Affiliations:** 1 Nursing Institute "Professor Radivoje Radić", Faculty of Dental Medicine and Health Osijek, Josip Juraj Strossmayer University of Osijek, Osijek, Croatia; 2 Faculty of Medicine, Josip Juraj Strossmayer University of Osijek, Osijek, Croatia; 3 University Hospital Centre Osijek, Osijek, Croatia; 4 General County Hospital Našice, Našice, Croatia; Universita degli Studi di Udine, ITALY

## Abstract

Core self-evaluation (CSE) is a theory that includes four personality dimensions: self-esteem, self-efficacy, locus of control and emotional stability. CSE proved to be a significant predictor of the research on cognitive, emotional and behavioral responses across various situations in the workplace. The aim of this study was to examine the relationship between personality traits of the core self-evaluation and clinical decision-making in nurses’ profession. A cross-sectional design was applied. Data was collected with standardized instruments: Core Self-Evaluation Scale and Clinical Decision-Making Nurses Scale, 584 nurses have participated in the study. Correlation and hierarchical regression analysis were used to test the relations and prediction of variables. The findings of the study revealed that there is a significant positive relationship between overall core self-evaluation and nurses’ clinical decision-making, and there is a significant contribution of self-esteem, self-efficacy and locus of control on all dimensions of clinical decision, especially in the area of canvassing of objectives and values. Nurses with high CSE have positive self-views and tend to be confident in their ability and they also feel in control while performing nursing interventions, whereas those with low CSE tend to have fewer accessible positive resources and are more prone to risk aversion.

## Introduction

Personality traits, as well as decision-making, show individual differences in people’s behaviour. The personality predisposes most of human functions, activities and interests. It is stated in numerous studies today, that decision-making in nursing practice is a complex process [1], but it is also an integral part of nursing profession [2, 3] and results from critical thinking [4]. Any clinical decision-making in nursing is directly under the influence of cognitive process and depends on the way nurses estimated the importance of received messages, their priorities and their capability of recognizing and responding to the often ambiguous clinical and non-clinical scenarios in the workplace. In addition to environmental factors that have been proven to influence clinical decision-making, only few studies examine the contribution of personality to decision-making, especially in the nursing area [5, 6, 7]. Theories that correlate human personality and emotions state that individuals have stable emotional styles or personality traits, emphasizing that personality can affect the expression of emotions [8]. Also, individuals attend to emotions as sources of information, and different types of emotion suggest different types of information [9]. In the absence of clear theoretical framework which links personality traits and emotional competence [10] in clinical decision-making, theoretical construct of this study is a kind of a dual process theory, described by a numerous psychologists [11, 12], which explains everyday decisions as based on a complex interplay between emotion-based and cognitive-based processing [13].

### Nurses clinical decision-making

In their everyday work, nurses make numerous decisions: ethical, clinical and practical [14]. Most decisions made by a nurse take place in an environment full of activities and conflicts [15]. Nurses’ decisions have a direct impact on the outcome of a health care as well as patients’ safety [16, 17]. Nurses’ decision-making has been examined using numerous methods and different theoretical approaches. The most common models used to describe nurses’ clinical decision making (NCDM) are the information-processing model, the intuitive-humanist model, and the multidimensional decision-making model [18]. The information-processing model assumes that thought processes follow rational logic and involve generating hypotheses and their subsequent interpretation and evaluation. The intuitive model is defined as “understanding without a rationale” [19] and relies mostly on nurses’ work experience. At the moment of making a decision, an experienced nurse uses patterns of similarity between the new and the familiar situation and identifies each subsequent step, instead of separating the situation in several segments [20]. NCDM as an information-processing model is associated with less experienced nurses or those who have just started working as nurses. In the education of nurses, the information-processing model is constantly emphasized. Namely, NCDM regularly monitors the nursing care process, which consists of assessment, diagnosis, planning, implementation and evaluation to make the clinical decisions effective [21]. Benner’s intuitive-humanist model is the opposite of the previous one [22]. Nurses with more experience rely on the sense of intuition. The development of professional expertise in nursing depends on the depth and range of clinical experience. Benner states that nurses with more than 5 years of work experience in one clinical area are developing an intuitive pattern for identifying patient needs, as well as determining the exact course of activity [23]. O’Neill et al. suggest that the complexity of clinical decision-making requires a broad knowledge base and access to reliable information sources as well as working in a supportive environment. Such multidimensional model involves the assembling of information into the thematic area; comparison of the advantages and disadvantages of the decision made and its alternatives; determining whether additional information is needed; and finally assessing which intervention is the most effective and justifiable [24]. In the nursing area, according to the Croatian health care delivery system, this would mean: collecting patient data, assessing their health status and identifying problems, analyzing the standard of nurses’ interventions and skills, risk assessment for selected interventions, recognizing environmental factors affecting decision-making, generating hypothesis and ultimately acting as a nurse. The variables that influence the multidimensional decision-making process consist of personal and environmental factors [25]. Concerning the environmental factors, most of the literature refers to the working conditions and characteristics of the environment, but also to the assessment of the severity of patient’s condition [26]. It is believed that personal characteristics may influence cognitive and emotional processes involved in decision-making [27].

### Core self evaluation

The “Core self-evaluation theory” by Judge and associates proved to be an excellent psychological construct for measuring personality traits and variables in organizational psychology. Core self-evaluation (CSE) represents the fundamental appraisals individuals make about their self-worth and capabilities. It represents stable personality construct, including individual subconsciousness and evaluation of one’s own abilities and self-control [28]. CSE includes four personality dimensions: self-esteem, self-efficacy, locus of control and emotional stability. Timothy Judge has developed the concept of ‘core self-evaluation’ in which he describes how individuals evaluate themselves, their attitude towards the environment and situations they find themselves in, as well as how they perceive their self-esteem and competences [29]. These four areas are not identical, each has its own contribution making a fundamental assessment of the individual. It was shown that this is such a powerful and highly consistent psychological construct which describes a significant connection between personality traits and pleasures, motivation and stress [30]. The basic premise is that individuals with high CSE value are considered to be skilled in carrying out activities at work and are prone to risk in decision-making situations because they use positive resources from their environment [31]. These persons are confident in their own abilities and feel control over the events. In the decision-making process, such person positively performs tasks without great stress and accepts to be the leader [32].

### Aim

With this study we wanted to examine whether the dimensions of personality traits could be implicated in decision-making strategies in the nursing area and, if so, in which direction the dimensions of personality contribute to the areas of nurses’ decision-making. Therefore, the aim of this study is to examine the correlation between personality traits of core self-evaluation and clinical decision-making in nurses’ profession.

## Materials and methods

### Procedure and participants

This study was conducted from April 2016 to January 2017, on a sample of 584 nurses who worked at University Hospital in Croatia, in the hospital wards, operating rooms, intensive care unit (ICU) or outpatient clinics. The study did not include respondents who were absent for a long period of time during data collection, due to either vacation or illness and those nurses who are not working in a direct contact with patients (department of sterilization, transfusiology, intrahospital infections, quality departments), as well as those who are leaders and are dealing with management rather than health care. The data was collected using survey questionnaires distributed by researchers (authors of this manuscript) to nurses in clinical wards at the time of their rest break. A total of 990 questionnaires were distributed. The response rate was 59% and the participants were selected using the principle of availability. 71 of all respondents immediately returned a non-completed questionnaire because they didn’t want to participate in the research. They have reason to say that they have no time or simply do not want to. Out of a total 619 collected questionnaires, 35 were excluded from further analysis because they were incomplete. The time to fill out the questionnaire was not limited, and it lasted averagely 20 minutes. In this study, 532 (91.1%) participants were female and 52 (8.9%) were male. 424 nurses (72.6%) had vocational diploma, 145 (24.8%) had Bachelor’s degree, and 15(2.6%) have Master’s degree. Considering working hours, 404 nurses (69.2%) had rotating 12-hour shifts and 132 nurses (22.6%) only worked 8-hour morning shift. The remaining 47 (8%) worked 8-hour daily shift (morning and afternoon).

### Measures

For the purposes of this research, two validated instruments were used: The Core Self-Evaluation Scale (CSES) and the Clinical Decision Making in Nursing Scale (CDMNS). For the purpose of selection, translation, validation and cross-cultural adaptation of the mentioned instruments, the following procedures were applied: (1) initial selection of instruments in accordance with the objective of the study, (2) obtaining permission from the authors to translate and apply CSES and CDMNS, (3) translation procedures and back-translation, (4) language proofreading of the Croatian version of the questionnaire and (5) evaluation of the statistical reliability and content validity of the questionnaires [33, 34]. The initial selection of the instruments was made by the authors of this article and two assistant professors in the field of nursing, in role of a professional committee formed for the purposes of this research. Consent for the translation and use of the CDMNS questionnaire was obtained from the competent university, George Mason University College of Health and Human Services, while the CSES questionnaire was free to use. The text of the instruments was translated into Croatian language by two independent translators, English professors employed at higher education institutions for nurses. Subsequently, the process of back-translation of both questionnaires into English was done by an independent English professor who did not participate in the primary translation of the questionnaires. The content of the Croatian versions of both questionnaires was confirmed by all the authors of this article and the professional committee. The first part of the questionnaire used in this survey contained questions about general data of respondents (age, sex, education level, years of work experience, working hours organized in shifts, etc.). The second part of the questionnaire consisted of CSES and CDMNS questionnaires is described below.

#### Core Self-Evaluation Scale (CSES)

The CSES is a 12-item questionnaire developed by Judge et al. to operationalize the construct of self-evaluation [35]. It has been designed to measure four traits that compose this construct: self-esteem (e.g. “Overall, I am satisfied with myself”), generalized self-efficacy (e.g. “I complete tasks successfully”), locus of control (e.g. “I do not feel in control of my success in my career”), and emotional stability (e.g. “Sometimes I feel depressed”). Items are rated on a scale ranging from 1 (“strongly disagree”) to 5 (“strongly agree”). The scale scores are the sum of the ratings of the items. Relevant items were reverse-coded. There has been good psychometric support for the CSES. Previously, it has been used in different samples and has demonstrated its reliability and validity [35]. In this study, after the aforementioned phases of the questionnaire preparation (translation, back-translation, language proofreading, expert analysis of content validity), the main research was conducted, on the basis of which the calculated values of Cronbach’s alpha were 0.74 for the total scale and for the subscales values were: 0.73 (self-esteem), 0.69 (generalized self-efficacy), 0.71 (locus of control), and 0.79 (emotional stability).

#### Clinical Decision Making in Nursing Scale (CDMNS)

The CDMNS is a 40-item questionnaire designed by Jenkins to operationalize the perception of the clinical decision making in nursing [36]. It assesses four dimensions: search for alternatives or options (e.g. “If the clinical decision is important and I have enough time, I will seek other alternative information and solutions”), canvassing of objectives and values (e.g. “I take into account the opinion of the patient, when I consider the decision to be made for him”), evaluation and reevaluation of consequences (e.g. “Before I make a decision, I think of all the possible consequences of that nursing intervention”), and search for information and unbiased assimilation of new information (e.g. “I solve the problem or make a decision without having to consult with someone”). Items were rated on a scale ranging from 1 (never) to 5 (always). Each subscale is composed of 10 items, 22 items are written as positive and 18 items are written as negative [36]. A high score indicates that the perception of decision making is high, whereas a low score indicates that the perception of decision making is low. The scale is evaluated through the scores obtained from each subscale and the total scale. The total score ranged from 40 to 200. The translated and adapted Croatian version of the CDMNS showed a high level of statistical confidence. Cronbach’s alpha was 0.94 for the total scale, and for the subscales values were: 0.93 (search for alternatives or options), 0.94 (canvassing of objectives and values), 0.95 (evaluation and reevaluation of consequences), and 0.94 (search for information and unbiased assimilation of new information).

### Ethical consideration

The Ethical Committee of University Hospital Centre Osijek (Approval number: R1-14772-2/2016) approved this research. All of the respondents were informed in writing about the aim of the research and signed an informed consent to participate in the research. The respondent’s anonymity, both during and after the research, was guaranteed.

### Data analysis

Numerical data was expressed as mean values (M) and standard deviation (SD). The correlation analysis evaluated, by Pearson’s correlation coefficient, the relationship between the areas of the CSE and the areas of the NCDM. In order to determine the relationship between variables, contribution of prediction variables (areas of CSE) to criterion (NCDM), a hierarchical regression analysis was used. All the statistical analysis were performed using the Statistical Package for Social Sciences (SPSS) version 15.0. (SPSS Inc., Chicago, IL, USA). In all the analyses p value < 0.05 is considered statistically significant.

## Results

### Descriptive statistics

The data presented in Table 1 show that a full range of answers were obtained for most subscales, which vouches for the good sensitivity of the instruments.

**Table 1:** Basic descriptive statistics for the measured variables.

Variables	Range	Mean	Standard deviation
Age	23–64	39.2	9.5
Length of service	1–45	19.0	9.6
CSES_total	24–56	40.5	5.3
CSES_emotional stability	3–15	6.9	2.0
CSES_locus of control	2–15	10.1	2.1
CSES_self-efficacy	6–15	11.5	1.5
CSES_self-esteem	6–15	10.8	1.6
CDMNS_total	75–190	135.8	27.6
CDMNS_alternative	15–49	33.6	9.6
CDMNS_ canvassing	19–49	36.6	7.3
CDMNS_ evaluation	17–49	35.1	9.1
CDMNS_information	17–49	33.9	8.9

CSES: Core Self-Evaluation Scale; CDMNS: Clinical Decision Making in Nursing Scale.

The average values of the subscales for both areas, core self-evaluation and clinical decision-making, lean slightly in the positive direction, except for the area of emotional stability (Table 1).

### Correlation analysis

The correlation analysis reveals significant positive correlations between total core self-evaluation and the overall clinical decision-making (r = .386; p< 0.001). (Table 2).

**Table 2:** Matrix of the intercorrelation of the measured variables.

**Variable**	**1.**	**2.**	**3.**	**4.**	**5.**	**6.**	**7.**	**8.**	**9.**	**10.**	**11.**
1. Length of service	1	-.071	-.021	-.070	-.052	-.064	.064	.210**	.321**	.060	.115*
2. CSES_total		1	.469**	.629**	.670**	.650**	.386**	.329**	.382**	.337**	.209**
3. CSES_emotional stability			1	-.258*	.249*	-.294*	-.255**	-.243**	-.315**	-.285**	-.207**
4. CSES_locus of control				1	.283**	.268**	.240**	.196**	.199**	.217**	.155**
5. CSES_self-efficacy					1	.631**	.369**	.365**	.392**	.355**	.241**
6. CSES_self-esteem						1	.383**	.352**	.390**	.338**	.349**
7. CDMNS_total							1	.690**	.871**	.888**	.810**
8. CDMNS_alternative								1	.406**	.431**	.322**
9. CDMNS_ canvassing									1	.800**	.694**
10. CDMNS_ evaluation										1	.671**
11. CDMNS_information											1

CSES: Core Self-Evaluation Scale; CDMNS: Clinical Decision Making in Nursing Scale.

Core self-evaluation is mostly associated with the area of canvassing of the objectives and values (r = .382; p< .001) and the lowest association is with the area of searching for information (r = .209; p< .001), although the result shows significance. From personality traits, the most associated with all areas of decision making is the characteristic of self-esteem (r = .383; p< .001). Emotional stability (r = -.255; p< .001) is the only characteristic in negative correlation with total CDMNS.

Furthermore, Table 2. shows significant and positive correlations between length of service and canvassing of the objectives and values (r = .321; p< .001), as well as search for alternatives or options (r = .210; p< .001).

### Regression analysis

The data clearly show (Table 3) an important contribution of core self-evaluation in the clarification of CDM in nurses, with 31.3% of total variance explained. In the first step, length of service, sex and age, did not make a big contribution to overall clinical decision-making. Statistically less significant, these variables still contribute to the area of clinical decision-making. Search for alternatives or options is explained with 5.5% variance, while canvassing of the objectives and values is explained with 4.6% variance.

**Table 3:** Regression analysis of the four criteria of core self-evaluation for the investigated variables.

Criteria/Predictors	search for alternatives or options	canvassing of objectives and values	evaluation and reevaluation of consequences	search for information and unbiased assimilation of new information	clinical decision- making nurses
	β	β	β	β	β
Sex	-.166	.015	-.029	.061	-.045
Age	-.148	.218	.048	.190	.075
Length of service	-.297	.211	.195	.261	.087
Regression model	R = .244;	R = .225;	R = .089;	R = .216;	R = .091;
	R^2^ = .059;	R^2^ = .051;	R^2^ = .008;	R^2^ = .046;	R^2^ = .008;
	R^2^_corr_ = .055;	R^2^_corr_ = .046;	R^2^_corr_ = .003;	R^2^_corr_ = .042;	R^2^_corr_ = .003;
	F_(3,580)_ = 7.06;	F_(3,580)_ = 3.51;	F_(3,580)_ = 2.88;	F_(3,580)_ = 5.38;	F_(3,580)_ = .572;
	p< .008	p< .061	p< .090	p< .021	p< .450
Sex	-.120	-.046	-.047	.006	-.066
Age	.127	.023	-.132	-.052	-.006
Length of service	-.297	.211	.195	.261	.087
CSES emotional stability	.097	.141	.160	.126	.095
CSES locus of control	.337	.321	.283	.213	.356*
CSES self-esteem	.328	.440	.396	.339	.460*
CSES self-efficacy	.100	.110	.098	.340	.264*
Regression model (final solution)	R = .516;	R = .568;	R = .483;	R = .438;	R = .567;
	R^2^ = .266;	R^2^ = .322;	R^2^ = .233;	R^2^ = .192;	R^2^ = .321;
	R^2^_corr_ = .257;	R^2^_corr_ = .314;	R^2^_corr_ = .224;	R^2^_corr_ = .182;	R^2^_corr_ = .313*;
	F_(7,576)_ = 4.32;	F_(7,576)_ = 5.65;	F_(7,576)_ = 3.9;	F_(7,576)_ = 0.1;	F_(7,576)_ = 4.2;
	p< .038	p< .018	p< .048	p< .997	p< .041

* β: beta values; p< 0.005; CSES: Core Self-Evaluation Scale.

In the second step of the hierarchical regression analysis, the areas of CSE are included. Self-esteem, with 46.0% of total variance explained, and locus of control, with 35.6% of total variance explained, are the best predictors of clinical decision making. Less accurate predictor, self-efficacy, with 26.4% of total variance explained, points to certain practical importance as well. The least accurate predictor was emotional stability, with 9.5% of total variance explained.

## Discussion

Research on the theory of self-evaluation conducted by Judge et al. has shown that this is a valuable psychological indicator, specifically related to job variables [30, 35]. However, self-evaluation theory has never been studied involving nurses as respondents, even though nurses make a large working population. Given the fact that decision-making in nursing is an important aspect of nursing work, with this research we wanted to examine how CSE contributes to better understanding of decision-making in nursing, especially can it anticipate decision-making strategies in the area of nursing. The results of our research confirms Judge’s theory, which states that “more is better” in terms of scale value [32]. Respondents who have high CSES values also have positive values in decision-making. However, we consider that such high CSE values are not always welcome, because they may lead to quicker decisions which are not necessarily more accurate [37]. In this research, nurses with a high CSE rating are confident in themselves and their own abilities and are convinced that their decisions will lead to positive results. They are not afraid to make decisions and do not worry about their negative and possibly bad outcomes because they are convinced that all troubles can be overcome, and all problems can be solved and corrected. They are convinced that there would not be any contingencies because everything is under their control. Even Judge himself points out that CSE is linked to working behavior, especially for employees who put greater value on personal independence and are associated with a sense of personal control [38]. However, too much confidence in own abilities leads to quick decisions without full data collection and analysis [39]. Otherwise, nurses with low CSE values select activities that have minimal potential hazards.

When analyzing CSE areas, especially self-esteem, which shows the most significant correlation and contribution to decision-making, we can point out that nurses with a high self-esteem assessment are convinced that they never make mistakes or that they are capable of making safe decisions without accepting opinion from anybody else. In this study, self-esteem has a major contribution to the area of NCDM—seeking alternatives and options. Respondents state that they always think about all possibilities before making a decision. They accept the ideas suggested by others and think about solutions that are not even available at the time of patient care. Ramanigopal found that there is a significant positive correlation between self-esteem and vigilant decision-making style, and a significant negative correlation between self-esteem and defensive avoidance style of decision-making [40]. In the case of risky NCDM, nurses with high self-esteem rely on their positive self-views and tends to be less defensive in response to a risky task, whereas low self-esteem nurses tend to have fewer accessible positive resources and thus are more prone to risk. Researches show that people with high self-esteem successfully face problems and tasks. They are optimistic and have positive emotions [41]. They are also cognitively more flexible, and two variables are important for them: work experience and cognitive strategies, which they have successfully developed during this experience [27].

Self-efficacy is characterized by belief in their own ability to successfully perform the tasks. Authors state that self-efficacy is the main attribute for decision-making [42, 43]. Nurses who assess themselves to be efficient in performing tasks take into account the possible consequences of nursing intervention and after making the decision and the interventions, think and evaluate the outcome of their outcome. In our study we can estimate that nurses with higher self-efficacy have developed sense of control, participate in treatment-related decisions and that they also assess the quality of health-related nursing care.

Dumitriu et al. determine the influence of the locus of control, as a CSE dimension, on decision-making, stating that the frequency and patterns of decision-making differ depending on whether the person is oriented to the external or internal locus of control [44]. Nurses oriented to the external locus of control, attribute their successes and failures to external forces, circumstances and actions of other people and are significantly compromised in their ability to make decisions professionally, thus making them less reliable in their nursing practice. In this research, nurses had developed more internal locus of control, meaning they were inclined to take responsibility for their actions and most often believed they are responsible for what is happening to them. In this research, the locus of control mostly contributes to the area of evaluation of the consequences in CDMN, which means that nurses think about the possible good and bad consequences of the intervention. They state that they make a decision based on the informations available and are aware of possible mistakes and responsibilities for the implemented actions as well.

When it comes to the area of emotional stability, nurses do not have high values of this trait, and emotional stability is negatively correlated with decision-making areas. This is consistent with the other results obtained in this research area, as respondents tend to be confident in their decisions and control in everyday work, they do not avoid making autonomous decisions, they are not inactive, indecisive [45, 46], nor cautious [41]. From the CSE area, self-esteem and self-efficacy have proven to be the predictors that best contribute to NCDM.

In addition, high NCDM values in all areas point to the fact that nurses have developed a critical thinking in everyday work while performing nursing interventions, regardless of the personality structure. Our research shows positive values in decision-making by most nurses and this result can only be attributed to nursing reforms in the Republic of Croatia where changes in nursing began with the establishment and work of the Croatian Chamber of Nurses in 2003. At that time, special regulations on professional development and the rights and responsibilities of nurses were issued [47]. After that, nurses increasingly took on the role of active decision-makers in health care, both by policy makers (nursing law) and other members of health care teams. More possibilities are created to strengthen the competence of nurses in clinical decision-making. The competence of nurses was for the first time defined by law in 2011. Educational requirements for the nurses have also increased since 2009, when the first university graduate program in nursing was established. A high awareness of the fact that nursing became independent and professional was created among nurses precisely because of the education development as well as the emergence of a scientifically based nursing [48]. There are many factors involved in NCDM and each of the factors has the potential to impact effective decision making. Knowledge, experience, work conditions and nurses’ personality have significant impact on the development of critical thinking skills [49]. Although the sample includes diverse nursing job positions, it originates from only one hospital. For further examinations, it is also recommendable to verify the five-factor model of personality and core self-evaluation. It is necessary to ascertain whether there are differences in instruments regarding cultural values of the respondents. It is also necessary to analyze a greater number of specialties in nursing. Conduction of longitudinal study would certainly be of a great significance.

## Conclusions

We conclude that personality traits are linked and that they contribute to clinical decision-making in nursing. Nurses with high values of self-esteem, self-efficacy, as well as the internal locus of control in performing their work make autonomous intervention decisions, seek alternative options, analyze patient data in detail, evaluate consequences of these interventions and partially seek new and additional informations. Moreover, because the decision-making process, particularly in nursing, is paramount to influencing patient treatment outcomes and safety, the practical implications of the conducted study are programs of lifelong learning and training which need to be developed to enhance the nurse’s skills in clinical decision-making. In addition, the distribution of nursing positions should be made according to the assessment of their personality traits because they affect the quantity and quality of their decisions, which can also affect the quality of work, job satisfaction and may lead to the increased retention rates in the healthcare workplace. On the other hand, the assessment of personality traits in the nursing students is not screening and professional selection, but helping them recognize how they react in certain situations, and through clinical education offer them opportunity to learn to cope with their emotions and make better decisions. The results of this study indicate the need for an early CSE empowerment interventions for nursing students in the earliest stages of their study. Thus, it is necessary to prepare students for work and decision-making in real-world clinical settings through a balanced effect on their cognitive and affective domains.

## Supporting information

S1 Dataset (XLS)
